# Giant Calcified Hepatic Hydatid Cyst: A Case Report

**DOI:** 10.7759/cureus.56876

**Published:** 2024-03-25

**Authors:** Anshu Sutihar, Deepak Lamichhane, Gubeanthrey JanakyRaman, Md. Mortuza Arafin, Rudish Jaz Shrestha, Niroj Pandey, Anil Yadav, Subash Uprety

**Affiliations:** 1 Internal Medicine, Dhaka Medical College Hospital, Dhaka, BGD; 2 Emergency Department, B. P. Smriti Hospital, Kathmandu, NPL; 3 Emergency Department, Hospital Tengku Ampuan Rahimah (HTAR), Klang, MYS

**Keywords:** echinococcus granulosus, helminth infection, infectious and parasitic diseases, role of imaging studies, cyst hydatid

## Abstract

Hydatid disease is a zoonotic disease caused by the parasite *Echinococcus granulosus*. It is an endemic disease in many parts of the world. Although humans are incidental hosts of the parasite, the disease sometimes results in fatal consequences. The liver and lungs are the most common sites of infection in humans. We report the case of a 45-year-old female who presented with complaints of right hypochondriac pain, fever, and cough, initially suspected as a case of liver abscess but later diagnosed as a giant calcified hydatid cyst of the liver. Imaging and immunoglobulin G for *Echinococcus granulosus* helped confirm our diagnosis. Based on her symptoms, the patient was treated symptomatically with analgesics, paracetamol, and an antitussive for pain, fever, and cough, respectively. In terms of definitive care, she was treated with oral albendazole and referred to her home district for necessary surgical intervention.

## Introduction

Hydatid disease is a parasitic disease caused by an infection from tapeworms called *Echinococcus granulosus* [1]. Humans are an accidental intermediate host during the parasite’s lifecycle. The right lobe of the liver is the most common site of hydatid cysts, where the ova is carried by the portal vein from the intestinal mucosa to the liver. Cysts may remain asymptomatic for many years before becoming evident, and most of the cases are diagnosed incidentally during abdominal pain workups [2]. The diagnosis of the hydatid cyst can be confirmed by imaging, serological tests, and immunological studies. The size, location, and extent of a hydatid cyst determine the therapeutic options. Treatment options for hydatid disease include surgery, chemotherapy, or a combination of the two. Chemotherapy is the most effective treatment when used in conjunction with surgery to prevent recurrence [3]. Complications arising from hepatic hydatid cysts fall into the following two main categories: rupture and subsequent bacterial infection. In 20-50% of the instances, the hydatid cyst ruptures, while secondary bacterial infection occurs in 5-8% of the cases [4]. In this article, we present the case of a 45-year-old woman presenting with symptoms mimicking a liver abscess, which was eventually diagnosed as a giant calcified hepatic hydatid cyst.

## Case presentation

A 45-year-old normotensive, non-diabetic female presented to the medicine department with complaints of right hypochondriac pain for one month, intermittent fever for three months, and cough for the same duration. The right hypochondriac pain was gradual in onset, mild to moderate in intensity, dull aching, and persisting for two to three hours without any radiation or associating factors. The patient described the fever as low-grade, intermittent, persisting for a few hours during the day, and subsiding with drenching sweat. It was not associated with chills and rigor. She also complained of a productive cough, which was whitish, scant, not foul-smelling, or mixed with blood, and more marked during the morning and evening.

The patient had a history of surgical intervention 12 years ago for a liver abscess when she presented with complaints of high-grade fever with chills and rigor and severe throbbing pain in the right hypochondriac region. She stated that dark brown-colored pus was aspirated, and, subsequently, a mass was removed from her abdomen.

On the general physical examination, she was ill-looking and moderately anemic. Other findings, including her vitals, were within the normal range. The abdominal examination revealed hepatomegaly about 8 cm from the right costal margin along the midclavicular line, which was tender to touch and firm in consistency with a smooth surface and regular margin. There was a right subcostal scar measuring about 3 inches from the previous surgery 12 years ago. Examination of the respiratory system revealed a dull percussion note, absent breath sounds, and vocal resonance in the right lower lung field from the seventh intercostal space downwards. Considering the medical history and examination findings, a provisional diagnosis of recurrent hepatic abscess was made.

Significant laboratory findings included a hemoglobin count of 7.5 g/dL, a hematocrit of 25.60%, a total white blood cell count of 12,000/mm^3^ with a neutrophil count of 70%, a mean corpuscular volume of 72.9 fL, mean corpuscular hemoglobin of 21.4 pg, a mean corpuscular hemoglobin concentration of 29.3 g/dL, an erythrocyte sedimentation rate (ESR) of 111 mm/hour, and a serum ferritin level of 10 ng/mL. The laboratory findings are shown in Table 1.

**Table 1:** Laboratory findings and reference values. MCV: mean corpuscular volume; MCH: mean corpuscular hemoglobin; MCHC: mean corpuscular hemoglobin concentration; ESR: erythrocyte sedimentation rate

Laboratory values	Patient values	Reference values
Total red blood cell count	3.51 million/μL	3.80–4.80 million/μL
Hemoglobin	7.50 g/dL	12–15 g/dL
Hematocrit	25.60%	34.80–45%
Total white blood cell count	12,000/mm^3^	4,000–11,000/mm^3^
Neutrophils	70%	40–80%
Lymphocytes	25%	20–40%
Monocytes	3%	2–10%
Eosinophils	2%	1–6%
Basophils	0%	<1–2%
MCV	72.9 fL	79–96 fL
MCH	21.4 pg	26–33 pg
MCHC	29.3 g/dL	32–36 g/dL
Platelets	450,000/mm^3^	150,000–450,000/mm^3^
ESR	111 mm/hour	0–12 mm/hour
Bilirubin	0.30 mg/dL	Up to 1.20 mg/dL
Alanine aminotransferase	10 U/L	Up to 40 U/L
Alkaline phosphatase	78 U/L	35–105 U/L
Fasting blood glucose	6.6 mmol/L	3.3–7.8 mmol/L
Serum creatinine	1.00 mg/dL	0.4–1.20 mg/dL
Serum ferritin	10 ng/mL	15–150 ng/mL
Sodium	138 mmol/L	135–140 mmol/L
Potassium	3.5 mmol/L	3.5–5.8 mmol/L
Chloride	101 mmol/L	95–107 mmol/L

**Figure 1:**
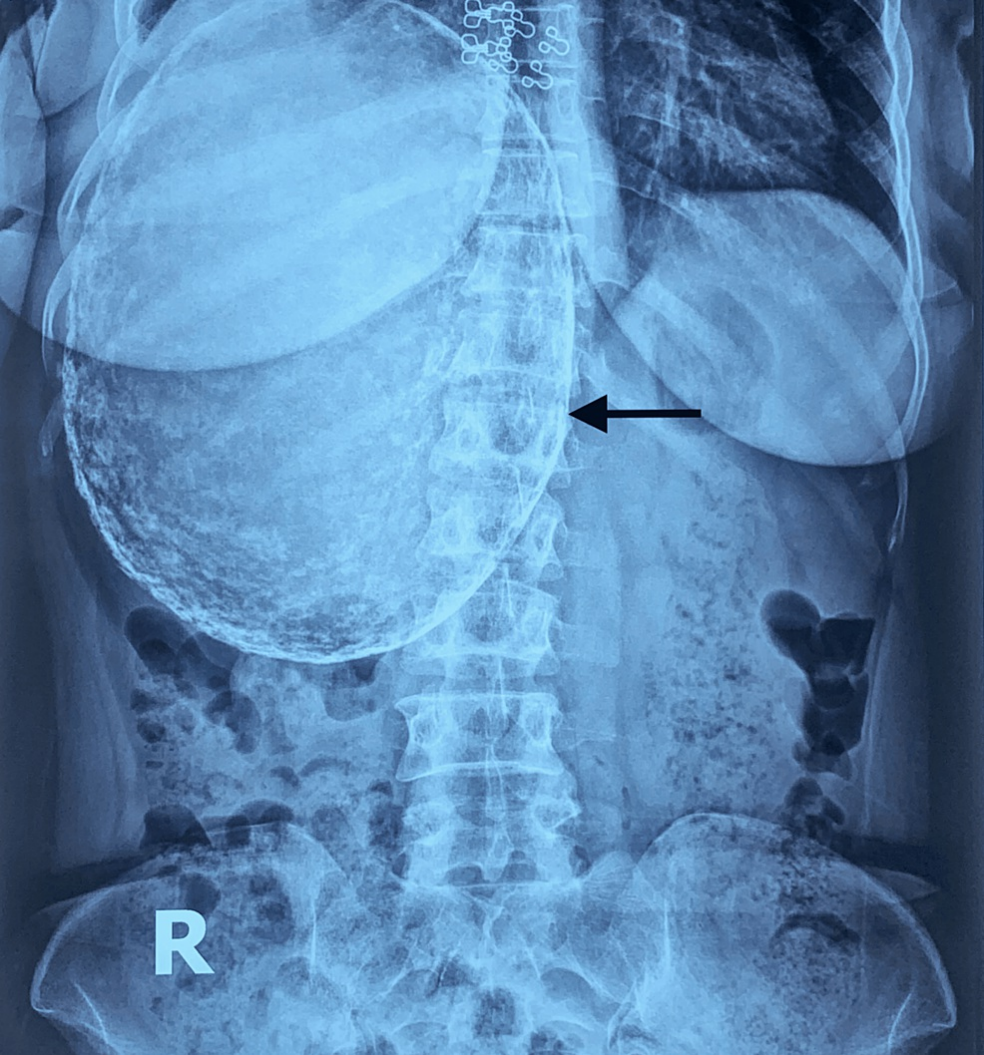
Plain X-ray of the abdomen. Plain X-ray of the abdomen in erect posture showing a huge, calcified cyst (black arrow) occupying the right hypochondriac and right lower chest regions.

Peripheral blood film report revealed microcytic hypochromic anemia with neutrophilic leukocytosis. A plain X-ray of the abdomen in an erect posture showed a huge, calcified cyst occupying the right hypochondriac and right lower chest regions (Figure 1).

**Figure 2:**
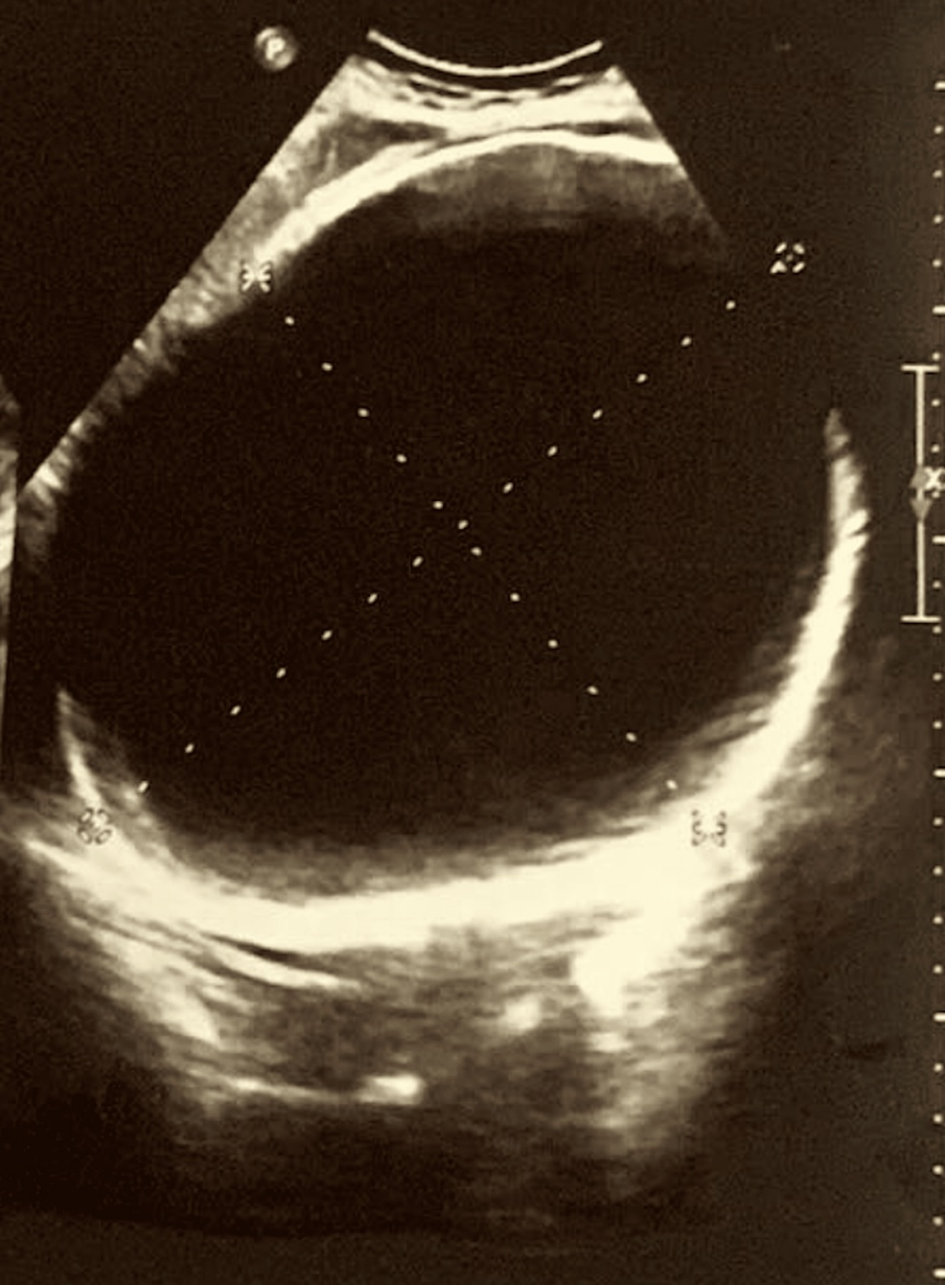
Ultrasonogram of the whole abdomen. Ultrasonogram of the whole abdomen showing a well-outlined clear fluid containing cyst measuring about 20 × 15 cm occupying the right hypochondriac region.

An ultrasonogram of the whole abdomen revealed a large cystic mass (20 × 15 cm) in the right hypochondriac region compressing the liver, whose origin could not be ascertained, likely hepatic/peritoneal (Figure 2).

**Figure 3:**
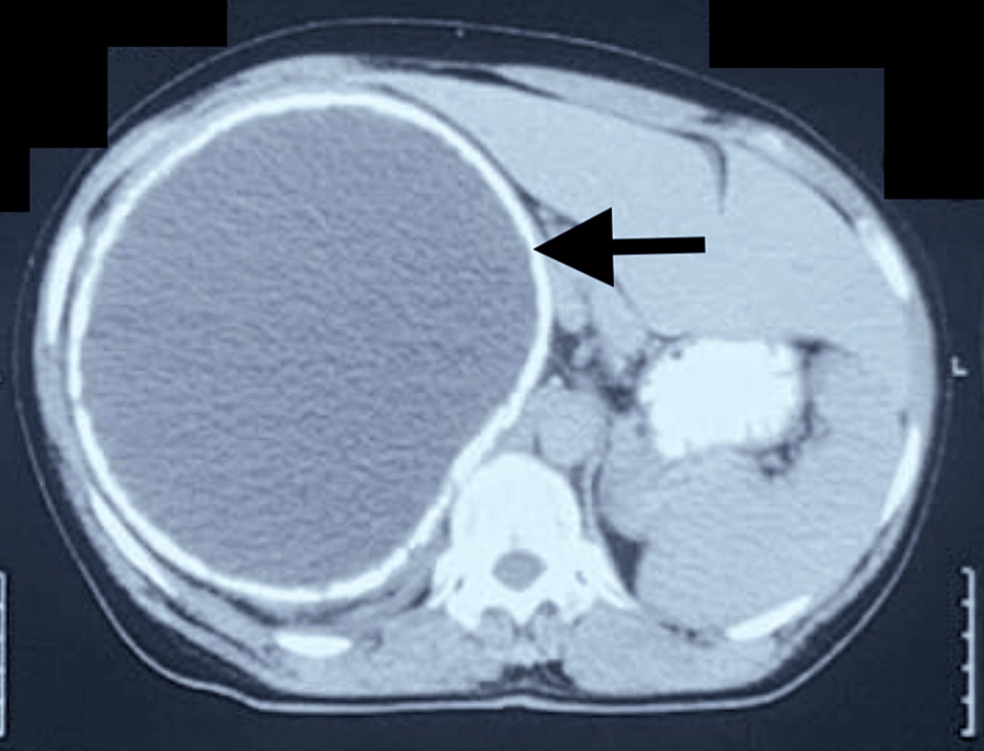
CECT scan of the abdomen (axial view). Axial CECT scan showing a large cystic lesion (black arrow) measuring about (18 × 15 cm) with a calcified rim in the right lobe of the liver. CECT: contrast-enhanced computed tomography

**Figure 4:**
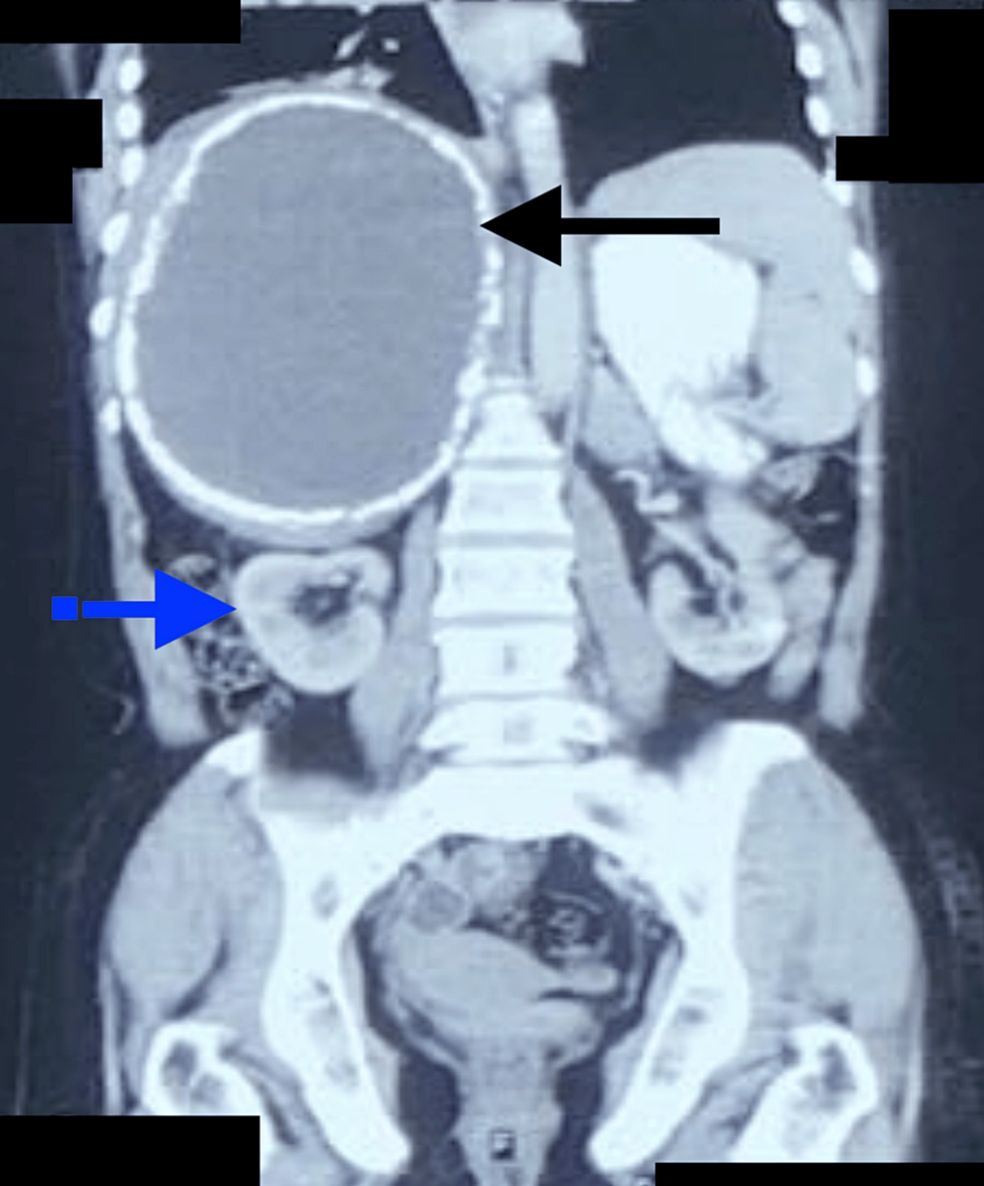
CECT scan of the abdomen (coronal view). Coronal CECT showing a large calcified cyst in the right lobe of the liver (black arrow), compressing the adjacent hepatic parenchyma and displacing the right kidney inferiorly (blue arrow). CECT: contrast-enhanced computed tomography

Contrast-enhanced computed tomography (CECT) scan of the abdomen showed a large cystic lesion measuring about (18 × 15 cm) with a calcified rim in the right lobe of the liver compressing the adjacent hepatic parenchyma. The liver was enlarged to a size of 22 cm, compressing and displacing the right kidney inferiorly (Figures 3, 4).

The scans pointed toward a diagnosis of a calcified hepatic hydatid cyst. To confirm this diagnosis, immunoglobulin G (IgG) for *Echinococcus granulosus* was done, which was positive.

The patient was treated symptomatically for pain, fever, and cough with analgesics, paracetamol, and an antitussive, respectively. Iron was infused to correct her anemia. She was started on oral albendazole 400 mg twice daily and was planned for surgery. Considering the patient’s request to shift to her home district due to personal preferences and her difficult financial situation to sustain expenses in another district, she was referred to her home district for necessary surgical intervention.

## Discussion

Hydatid disease, which affects the human species, is a parasitic infection caused by the larval stage of *Echinococcus granulosus*. Dogs are the definitive host where the parasite reproduces in the gut and releases eggs in their feces. Sheep, cattle, and horses are some intermediate hosts that graze on grass contaminated by the eggs of the parasite. Humans are the accidental intermediate hosts, as they can get infected by handling or ingesting food, water, or soil contaminated with stool from infected dogs [5]. Ingested eggs release larvae called oncospheres that are capable of penetrating the intestinal wall of humans and reaching the portal venous circulation. Through the portal venous system, oncospheres can get access to the liver, lungs, and various other organs [6].

Patients with hydatid cysts can present with various features according to the size and location of the cysts. Vomiting, discomfort, pain in the epigastrium, and weight loss are the common presentations [7]. If the cyst involves the lungs, it can result in chronic cough, dyspnea, pleuritic chest pain, and hemoptysis [6]. Rarely, patients may present with transient neurological deficits if the brain is involved, and features of obstructive jaundice are seen if the cyst invades the pancreas [5]. The hydatid cysts in the liver, when not treated by appropriate interventions, may lead to fatal complications, most commonly, rupture of the cyst in the intrabiliary channels. The cyst may also rupture into the peritoneal or thoracic cavity and may give rise to secondary bacterial infections [4].

The complete diagnosis of the hydatid cyst is made by imaging studies such as an ultrasonogram, computed tomography (CT), and magnetic resonance imaging, as well as serological and immunological studies. The first imaging technique to diagnose the hepatic hydatid cyst is an ultrasonogram. However, a CT scan is more accurate and is helpful in easily detecting the calcifications within the cysts [3]. When the imaging studies are not enough to reach a specific diagnosis, then immunological and serological studies are used as supplementary diagnostic measures to confirm the diagnosis. In cases where the diagnosis is not conclusive by these measures, fine-needle aspiration of the cystic fluid or biopsy are the measures to be used [8]. To confirm the diagnosis in our case, IgG against *Echinococcus granulosus* was done after a plain X-ray, ultrasonogram, and CECT scan were suggestive but not conclusive for the diagnosis of hydatid cyst.

There are multiple approaches to the management of hydatid cysts, such as chemotherapy, surgery, therapeutic endoscopic intervention, and minimally invasive therapies such as puncture-aspiration-injection-reaspiration, puncture-aspiration-injection-reaspiration-drainage, the modified catheterization technique, and percutaneous evacuation. Medical management with albendazole or mebendazole is considered appropriate for small and inoperable hydatid cysts [9]. The gold standard therapy for hydatid cysts is surgery that may or may not involve the resection of the pericyst, such as a simple cystectomy, partial pericystectomy, or even hepatectomy. However, endoscopic interventional methods such as stenting, sphincterotomy, or endoscopic retrograde cholangiopancreatography can also be used. Minimally invasive therapies have high applicability and fewer complications for patients not responding to drug therapies and with surgical contraindications [10].

## Conclusions

Cystic hydatid disease of the liver is a slow-progressing disease that may remain asymptomatic for long periods, and, on presentation, the symptoms are not specific. This makes clinical diagnosis difficult. A high degree of clinical suspicion is required to reach the definitive diagnosis early, as its presentation could be vague and may sometimes mimic a liver abscess. Hence, in the event of a cystic lesion of the liver, one must consider hydatid disease of the liver as one of the differentials. This is to prevent fatal complications such as rupture of the cyst, as the disease can be identified early with appropriate imaging, serologic, and immunologic modalities and treated promptly with simple anthelmintic therapy, minimally invasive therapies, or surgery if required.
